# Vaginal microbiota signatures in healthy and purulent vulvar discharge sows

**DOI:** 10.1038/s41598-022-13090-8

**Published:** 2022-06-01

**Authors:** André P. Poor, Luisa Z. Moreno, Matheus S. Monteiro, Carlos E. C. Matajira, Maurício C. Dutra, Diego F. Leal, Ana Paula S. Silva, Vasco T. M. Gomes, Mikaela R. F. Barbosa, Maria Inês Z. Sato, Andrea M. Moreno

**Affiliations:** 1 Department of Preventive Veterinary Medicine and Animal Health, School of Veterinary Medicine and Animal Science, University of São Paulo, Av. Prof. Dr. Orlando Marques de Paiva 87, Sao Paulo, SP 05508-270 Brazil; 2 Santo Amaro University (UNISA), R. Prof. Enéas de Siqueira Neto 340, Sao Paulo, SP 04829-300 Brazil; 3 Facultad de Ciencias Básicas, Universidad Santiago de Cali, Calle 5 #62-00, Cali, Colombia; 4 College of Veterinary Medicine, Department of Population Health and Pathobiology, North Carolina State University, Raleigh, NC 276607 USA; 5 Environmental Company of the State of São Paulo (CETESB), Av. Prof. Frederico Hermann Júnior 345, Sao Paulo, SP 05459-900 Brazil

**Keywords:** Microbiology, Applied microbiology, Bacteriology, Microbial communities, Infectious-disease diagnostics, Pathogens

## Abstract

Purulent vulvar discharges, primarily caused by genito-urinary tract infections, are an important source of economic loss for swine producers due to sow culling and mortality. However, the agents that compose the vaginal microbiota of sows and their changes during infections are not well understood. The first goal of this study was to characterize and compare the vaginal bacterial content of healthy (HE, *n* = 40) and purulent vulvar discharge sows (VD, *n* = 270) by a culture-dependent method and MALDI-TOF MS identification. Secondly, we performed 16S rRNA targeted metagenomic approach (*n* = 72) to compare the vaginal microbiota between these groups. We found a wide variety of bacteria, with *Proteobacteria, Firmicutes,* and *Bacteroidota* being the most abundant phyla in both groups, as well as *Escherichia-Shigella, Streptococcus,* and *Bacteroides* at the genus level. Most agents identified in the sequencing method also grew in the culture-dependent method, showing the viability of these bacteria. Alpha diversity did not differ between HE and VD sows, regarding sample richness and diversity, but a beta-diversity index showed a different microbiota composition between these groups in two tested herds. ANCOM analysis revealed that *Bacteroides pyogenes* were more abundant in VD females and can be a marker for this group. Other agents also require attention, such as the *Streptococcus dysgalactiae* and *Staphylococcus hyicus* found in remarkably greater relative abundance in VD sows. Network analysis revealed important positive correlations between some potentially pathogenic genera, such as between *Escherichia-Shigella*, *Trueperella*, *Streptococcus*, *Corynebacterium,* and *Prevotella*, which did not occur in healthy sows. We conclude that the alteration of the vaginal microbiota between healthy and purulent vulvar discharge sows, although not extreme, could be due to the increase in the relative abundance of specific agents and to associations between potentially pathogenic bacteria.

## Introduction

Purulent vulvar discharges caused by bacterial infections are a compelling cause of the decreased reproductive performance of sows^1–4^. Metritis and endometritis are the most common cause for these purulent discharges^1^ and are an important source of sow culling and mortality, which is increasing worldwide^2^. Infectious agents causing these conditions have been identified by traditional methods that rely on bacterial culture and isolation^3–6^, but few studies have characterized the microbiota in the vaginal canal of sows by high-throughput sequencing methods^7–10^, and less so compared between healthy sows and those with vulvar discharge^11,12^.

Previous studies show that the vaginal microbiota can play a significant role as a biological barrier against disease, due to its constitution and secretion of antimicrobial components^13–15^. Thus, a better understanding of the bacterial vaginal composition in healthy and diseased sows has the potential to mitigate urogenital infections with more specific treatments and better prevention strategies, improving sow welfare and reducing economic losses.

Wang et al.^11^ characterized the vaginal microbiota of sows with and without endometritis and found higher abundances of *Escherichia-Shigella, Bacteroides, Fusobacterium,* and *Clostridium_sensu_stricto_1* genera in diseased than in healthy sows, suggesting that the microbiota of these groups could be different. Zhang et al.^12^ investigated the effect of intestinal microbiota on vaginal bacterial composition in sows and indicated that some genera such as *Escherichia-Shigella* and *Bacteroides* may be linked to the onset of endometritis. However, Wang et al.^11^ did not perform a differential abundance analysis test and both studies evaluated a small number of females (*n* = 8) and should be of limited value. Our study hypothesizes that the vaginal microbiota of sows with and without vulvar discharge may present differences in bacterial composition. Therefore, we evaluated 310 vaginal samples by culture and MALDI-TOF (Matrix-Assisted Laser Desorption Ionization Time-of-Flight) mass spectrometry identification, and 72 of these samples were further characterized by targeted metagenomic sequencing to compare the vaginal microbiota between healthy and purulent vulvar discharge sows.

## Results

### Culture and MALDI-TOF MS identification

Bacterial culture and identification by MALDI-TOF mass spectrometry were able to identify a wide variety of species in sows presenting vulvar discharge. Of the 270 females with this clinical sign, 98.5% (266/270) were positive for bacterial isolation. Eighty-nine percent of the samples generated mixed cultures, with up to eight distinct species isolated from the same animal. For the 40 healthy females, only one did not present bacterial growth and 82% (32/39) of the samples generated mixed cultures.

**Figure 1:**
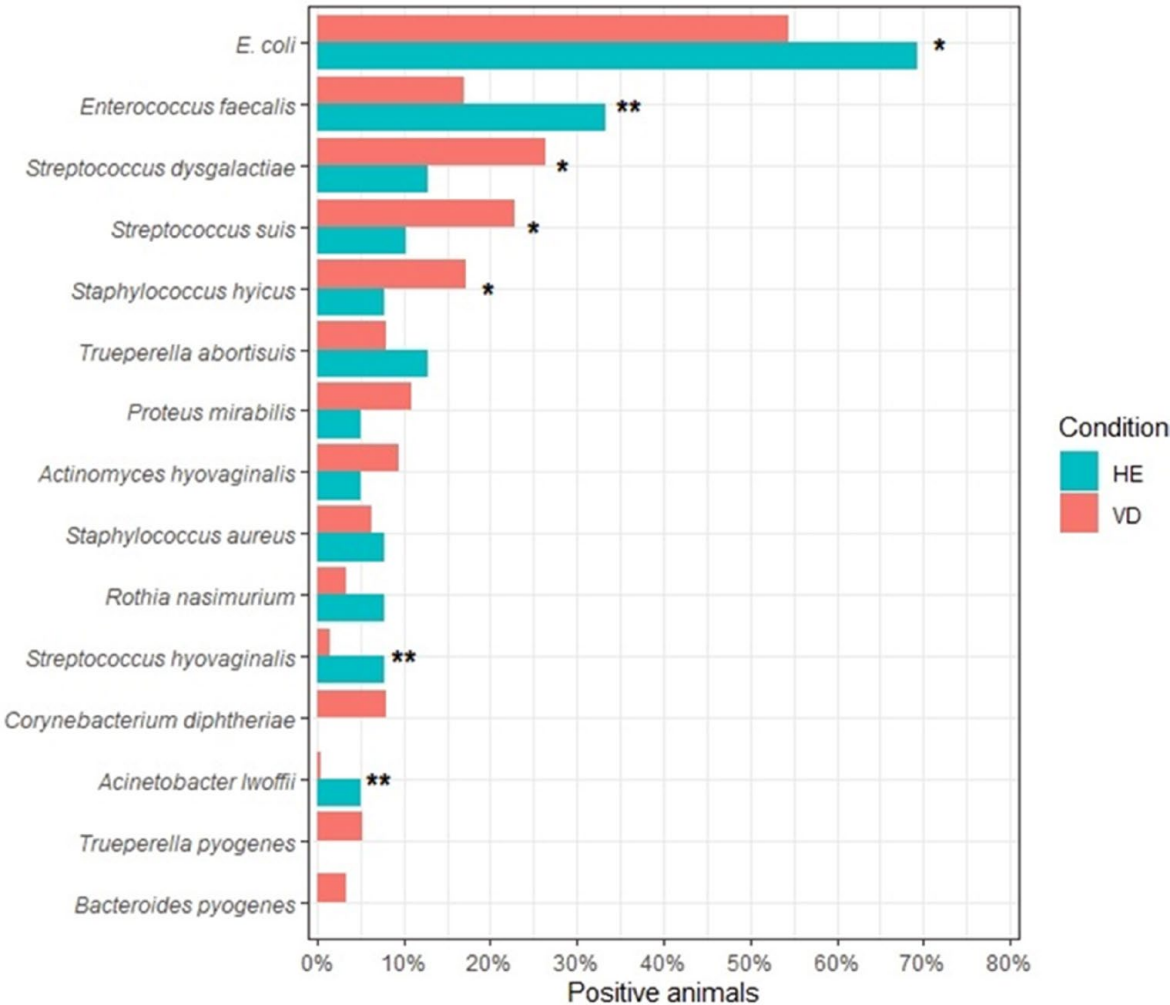
Frequency of the main species identified in HE and VD female samples in the culture technique and identification by MALDI-TOF MS. Red: sows with purulent vulvar discharge (VD); Blue: healthy sows (HE). *Fisher Exact Probability Test, 0.05 < *p* < 0.1. **Indicates significant differences between  groups (Fisher Exact Probability Test, *p* < 0.05).

As there was no statistical difference between the frequencies of the main agents for the different herds in the culture method (Supplementary Table S1), we analyzed the herd data together. The culture method showed that HE sows had a higher frequency of some bacterial species than VD sows (Fig. 1), such as *Enterococcus faecalis* (33.3% vs. 16.9%, *p* = 0.02), *Streptococcus hyovaginalis* (7.7% vs. 1.5%, *p* = 0.04) and *Acinetobacter Iwolffii* (5.1% vs. 0.4%, *p* = 0.04). Although HE sows had a higher frequency of *Escherichia coli*, we observed no statistically significant difference between groups (*p* = 0.06). VD sows also showed high frequencies for some species, but also without significant difference, such as *Streptococcus dysgalactiae* (26.3% vs. 12.8%, *p* = 0.09) and *Staphylococcus hyicus* (17.3% vs. 7.7%, *p* = 0.09). *Trueperella pyogenes* (5.3% vs. 0%), *Bacteroides pyogenes* (3.4% vs. 0%) and *Corynebacterium diphtheriae* (7.9% vs. 0%) were only isolated in VD sows.

**Figure 2:**
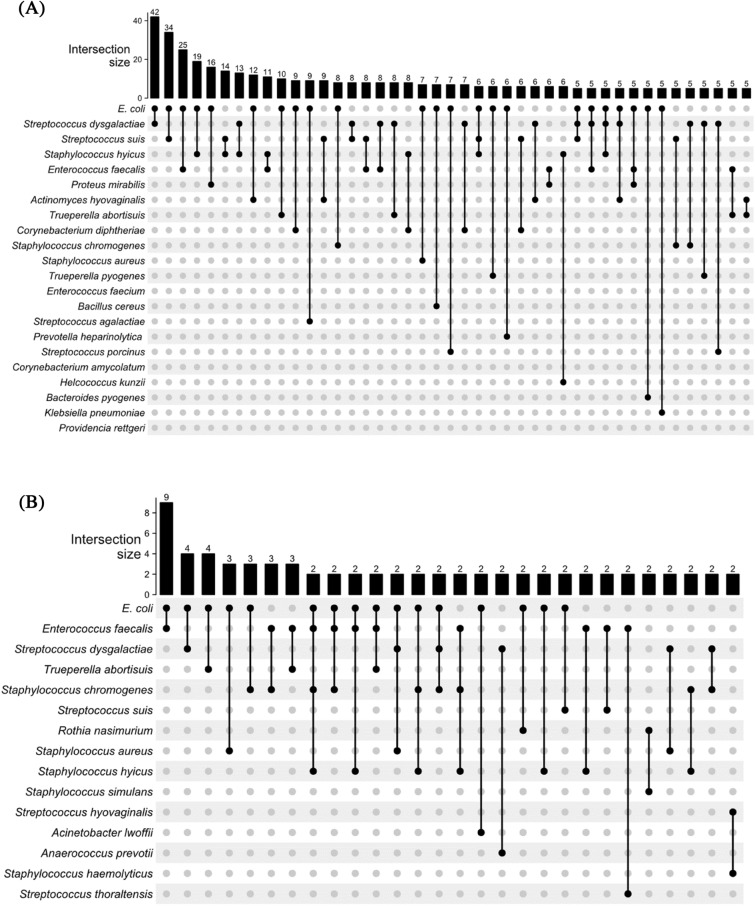
UpSet plot for (**A**) animals with vulvar discharge (VD; *n* = 266) and (**B**) UpSet plot for healthy animals (HE, *n* = 39). The numbers on the bars show the number of animals in which the agents, indicated by the filled dots, were isolated together.

**Figure 3:**
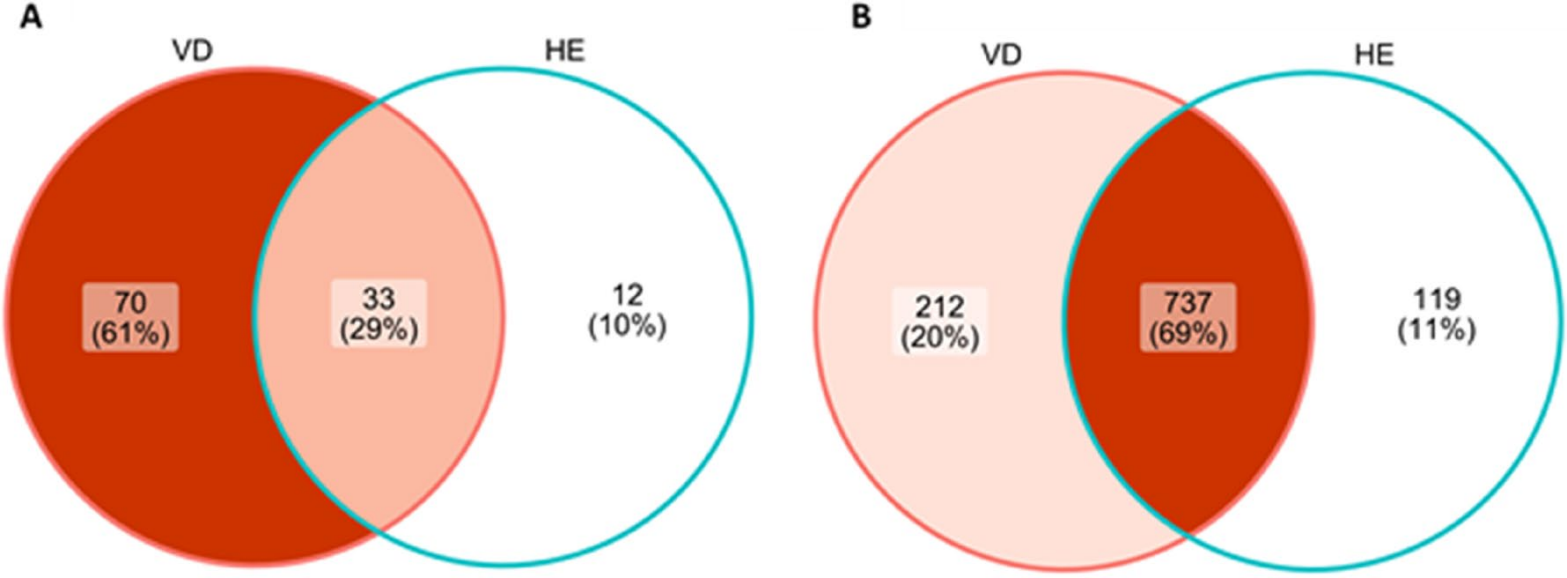
Venn diagram for (**A**) MALDI-TOF mass spectrometry bacterial identification and (**B**) OTUs between sows with purulent vulvar discharge (VD) and healthy (HE) sows.

Figure 2 shows an UpSet plot for the most common bacterial associations that occurred in these groups. VD sows showed more associations between species, with the most frequent one being *E. coli* and *S. dysgalactiae*, with 42 animals (15.8%). This was followed by *E. coli* and *Streptococcus suis* (12.8%), *Escherichia coli* and *E. faecalis* (9.4%), *E. coli,* and *S. hyicus* (7.1%). Meanwhile in HE sows, *E. coli* and *E. faecalis* (23%), *E. coli* and *S. dysgalactiae* (10.2%), *E. coli* and *Trueperella abortisuis* (10.2%) were the most frequent associations. Furthermore, the Venn diagram generated for the culture and MALDI-TOF MS identification results (Fig. 3A) shows that of the 115 bacterial species identified, 61% were exclusive to VD sows, 33% shared between VD and HE, and only 12% were exclusive to HE.

### Targeted metagenomic sequencing

We analyzed 72 samples by 16S rRNA gene metagenomic sequencing and a total of 3,760,359 raw reads were obtained. The median number of reads per sample was 39,233, with a maximum of 216,213 and a minimum of 3276. After sequence quality control, denoising, and clustering of amplicons at 97% identity, a total of 1,068 OTUs were obtained from the 72 samples (40 from vulvar discharge sows and 32 from healthy sows).

### Alfa and beta diversity analysis

A primary analysis to compare bacterial diversity among herds showed a difference for alpha diversity indices. Herds 1 and 3 had higher richness compared to herds 2 and 4 (*p* < 0.001, Chao1 index, see Supplementary Fig. S1 online). However, only herd 3 had higher diversity compared to all other herds (*p* < 0.01, Shannon index). There was also a difference in the beta-diversity analysis comparing herds. In the UniFrac PCoA analysis (see Supplementary Fig. S2 online), only herd 1 and 2 did not show a significant difference from each other, while the other pairwise PERMANOVA results (herd-1 vs. herd-3; herd-1 vs. herd-4; herd-2 vs. herd-3; herd-2 vs. herd-4; herd-3 vs. herd-4) indicated significant differences between bacterial community (*p* < 0.05 and *q* < 0.05).

**Figure 4:**
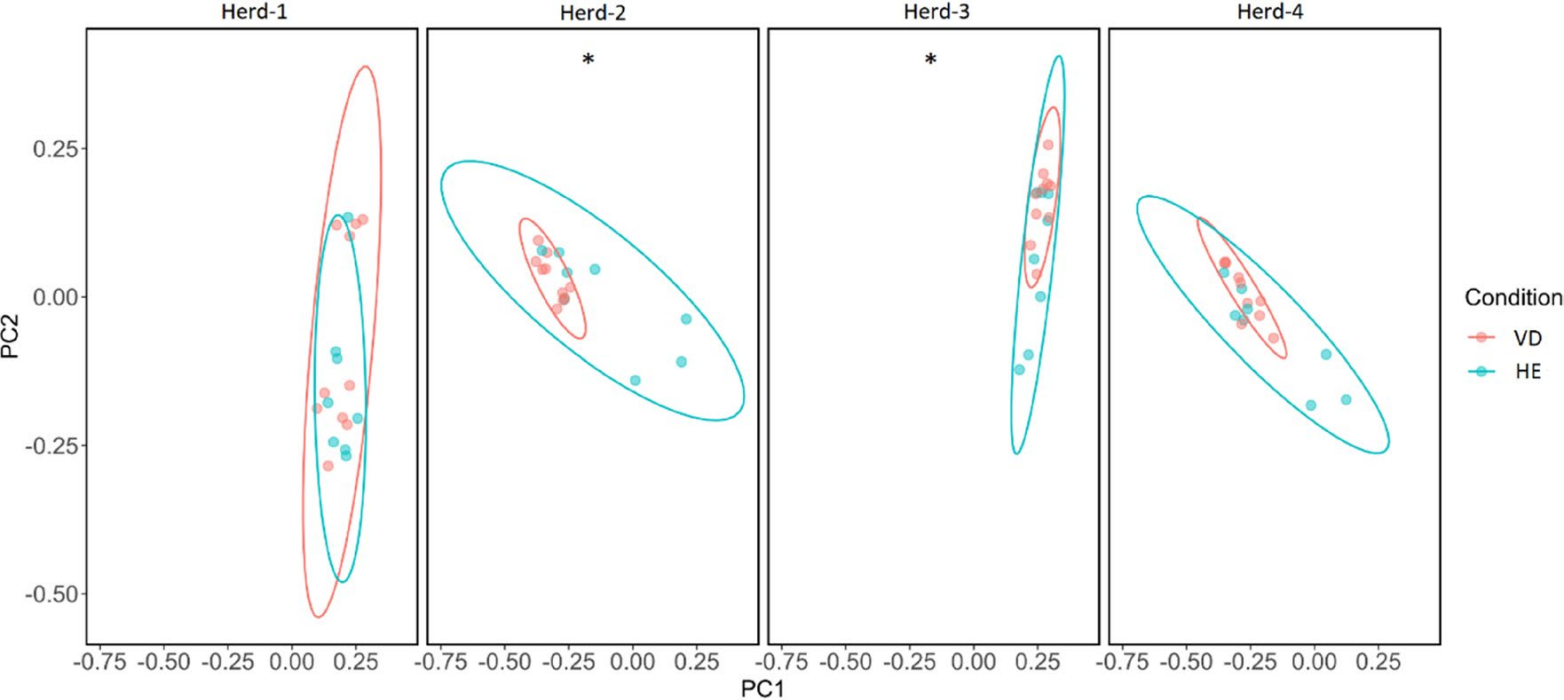
Unweighted UniFrac Principal Coordinates Analysis (PCoA) showing the clustering between sows with or without purulent vulvar discharge for each herd. Red: sows with purulent vulvar discharge (VD); Blue: healthy sows (HE). * Indicates significant differences between VD and HE (PERMANOVA, *p* < 0.05; *q* < 0.05).

To analyze diversity between groups of sows with and without purulent vulvar discharge, samples were analyzed separately for each herd. There were no differences between these groups for Chao1 and Shannon indices regarding community richness and diversity. The Venn diagram also shows that VD and HE share the most OTUs (69%) (Fig. 3B). For the beta diversity analysis, the PCoA based on Unweighted UniFrac and PERMANOVA analysis showed that for herds 2 and 3 there was a difference between HE sows and VD sows (*p* < 0.05, *q* < 0.05). For herd 4 there was a tendency for difference between clusters (*p* = 0.064, *q* = 0.064) (Fig. 4).

### Taxonomy composition and differential abundance analysis

**Figure 5:**
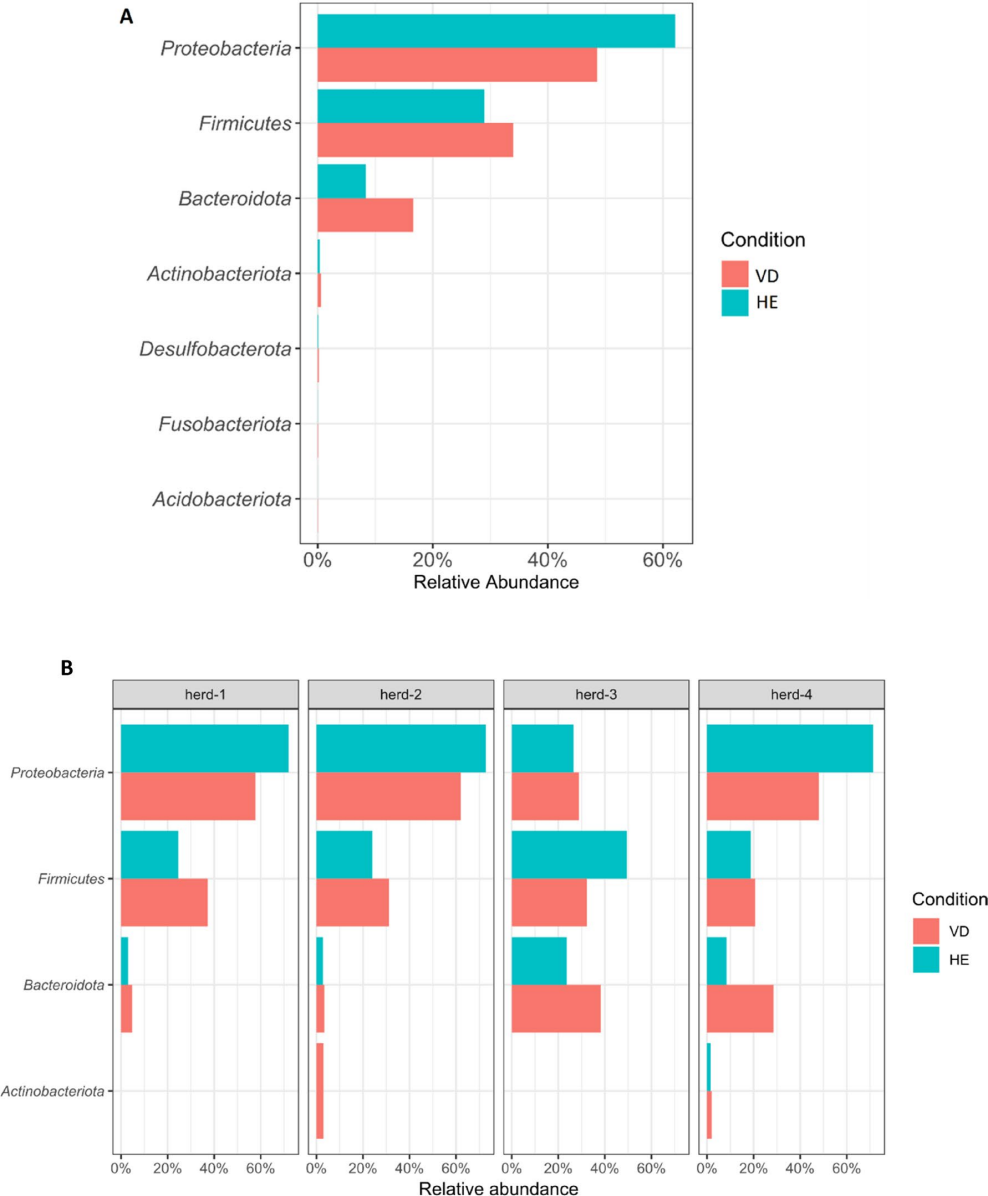
Relative abundance of the most influential phyla in bacterial vaginal microbiota of sows for the entire dataset (**A**) and each herd (**B**). Red: sows with purulent vulvar discharge (VD); Blue: healthy sows (HE).

Analyzing the herds together at the phylum level, *Proteobacteria* had the highest relative abundance in healthy and vulvar discharge sows, which accounted for 62.1% and 48.2% of relative abundance, respectively, followed by *Firmicutes,* which accounted for 29% and 34% of relative abundance, respectively, and *Bacteroidota,* which accounted for 8.4% and 16.6% of relative abundance, respectively (Fig. 5A). These three phyla represented a bacterial vaginal core for both groups since the other phyla identified represent less than 1% of relative abundance.

When herds were analyzed separately, some differences in relative abundance were observed (Fig. 5B). A higher abundance of *Proteobacteria* was observed for herd-1 (HE: 72%, VD: 57%) and herd-2 (HE: 72.8%, VD: 62.1%) with a lower relative abundance of *Bacteroidota* (HE: 3%, VD: 4.77%; HE: 2.85%; VD: 3.44%). In contrast, herd-3 had a lower relative abundance of *Proteobacteria* (HE: 26.5.6%, VD: 28.9%) but a higher relative abundance of *Bacteroidota* (HE: 23.6%, VD: 38.2%). Herd-3 still had a higher relative abundance of *Firmicutes* (HE: 49.5%, VD: 32.2%) compared to other herds. Herd-4 had a high relative abundance of *Proteobacteria* (HE: 71.3%, VD: 48%) and *Bacteroidota* (HE: 8.38%, VD: 28.6%), but a lower relative abundance of *Firmicutes* compared to other herds (HE: 18.8%, VD: 20.7%). Except for herd-3, the relative abundance of *Proteobacteria* was higher in HE females than in VD females in all herds. Conversely, the phylum *Bacteroidota* was higher in VD females in all herds, especially in herd-3 and herd-4.

**Figure 6:**
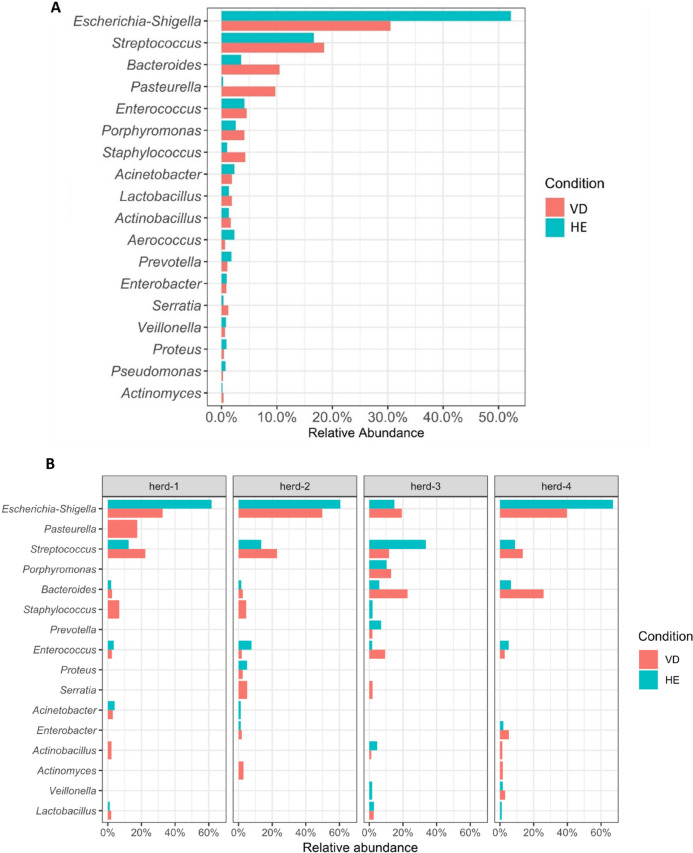
Relative abundance of the most influential bacterial genera in bacterial vaginal microbiota for the entire dataset (**A**) and each herd (**B**). Red: sows with purulent vulvar discharge (VD); Blue: healthy sows (HE).

At the genus level (Fig. 6A), *Escherichia-Shigella* was the most abundant in both groups but was higher in healthy than in vulvar discharge sows, accounting for 52.2% and 30.5% of relative abundance when data from all herds were analyzed together. *Acinetobacter*, *Aerococcus*, *Prevotella,* and *Pseudomonas* also showed a slightly higher relative abundance in healthy sows. Conversely, *Streptococcus*, *Bacteroides*, *Pasteurella, Porphyromonas,* and *Staphylococcus* were more abundant in sows with purulent vulvar discharge. For *Pasteurella*, however, all sequences came from a single sample in the VD group.

The graph in Fig. 6B shows the genera with more than 1% of relative abundance for each herd. *Escherichia-Shigella, Streptococcus,* and *Bacteroides* remain the main genera, present in all herds. But remarkable differences can be seen for *Porphyromonas* in herd-3, *Bacteroides* in herd-3 and herd-4, and *Staphylococcus* in herd-1 and herd-2.

**Figure 7:**
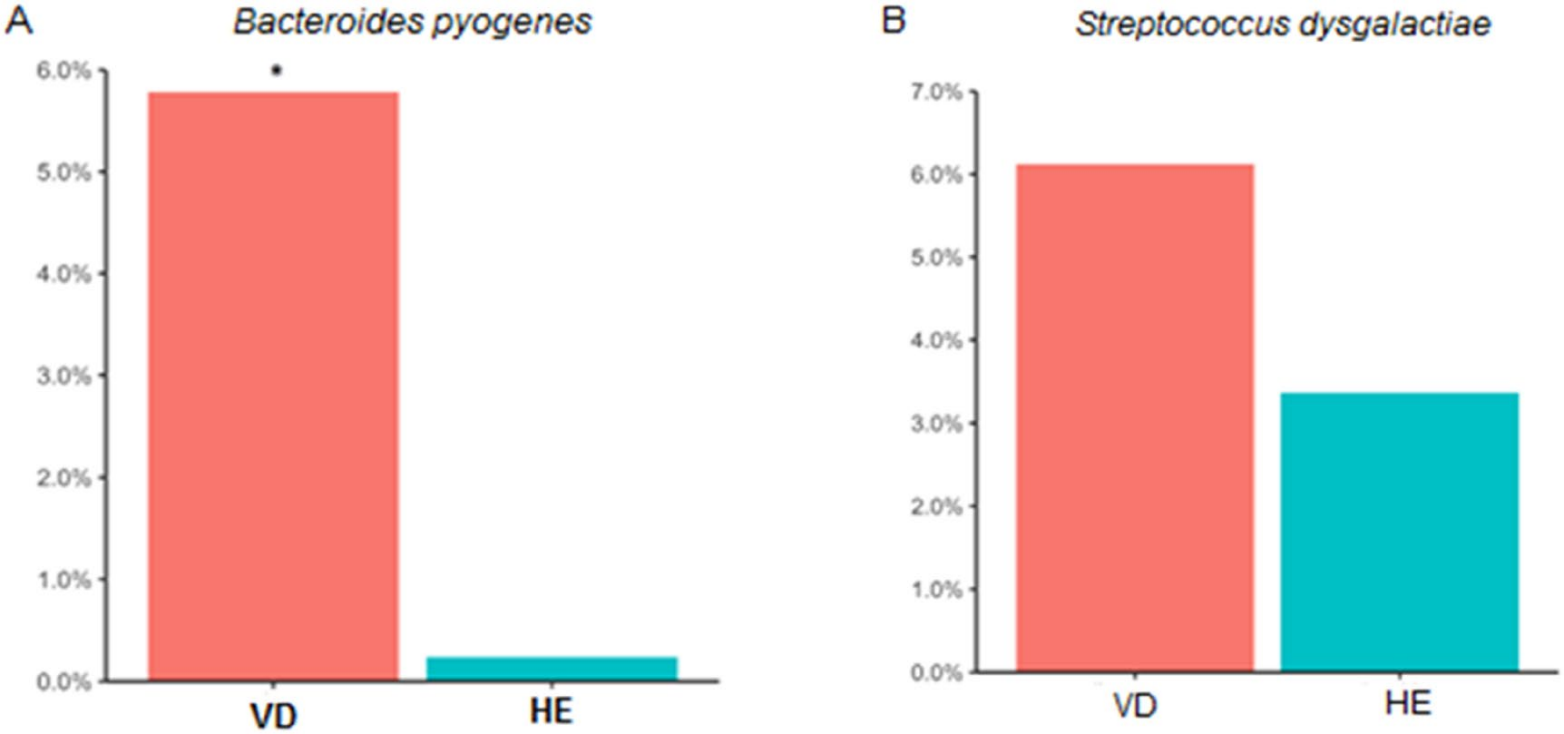
Relative abundances between VD and HE sows for (**A**) *B. pyogenes* and (**B**) *S. dysgalactiae*. Red: sows with purulent vulvar discharge (VD); Blue: healthy sows (HE). *Indicates significant differences between groups by ANCOM test (W = 44).

Since some OTUs were able to be classified at the species level, it is noteworthy that *B. pyogenes* and *S. dysgalactiae* were found in remarkable greater relative abundance in females with purulent vulvar discharge than in healthy females (Fig. 7).

The differential abundance analysis performed by the ANCOM method between VD and HE females across the entire dataset revealed a significant difference for the species *B. pyogenes*, with greater relative abundance in VD females (W = 44, Supplementary Table S2). When the test was applied to each farm separately, *B. pyogenes* were significantly more abundant in VD females only in herd-3 (W = 632, Supplementary Table S3). *S. dysgalactiae* was also found in greater abundance in VD females (Fig. 7). Interestingly, this difference was not statistically significant by the ANCOM test.

### Network analysis

**Figure 8:**
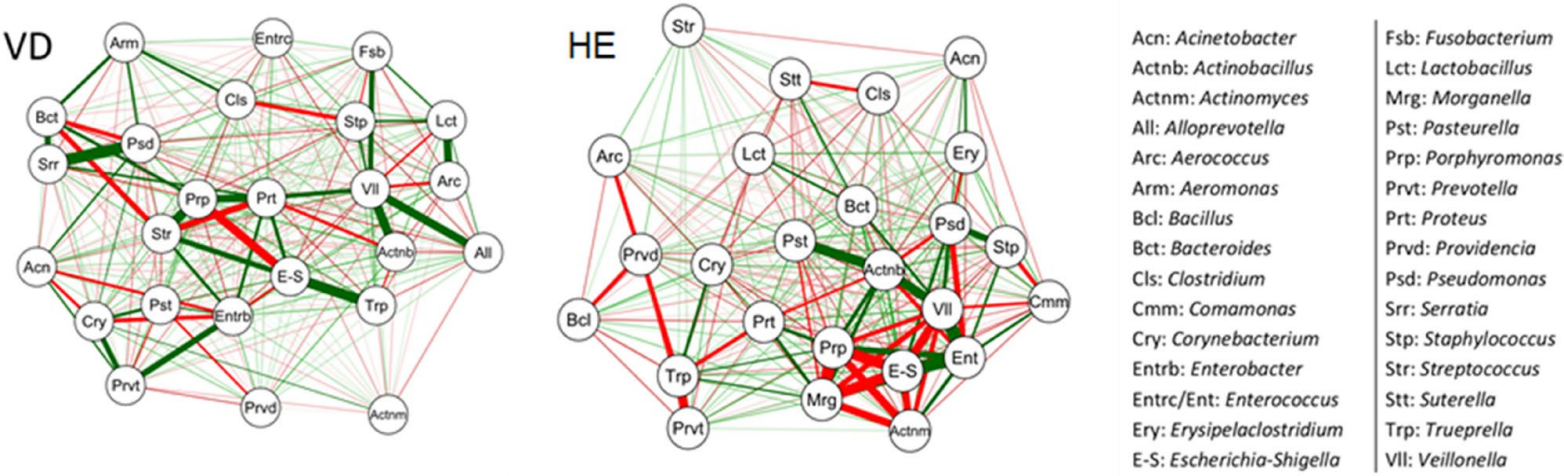
Network analysis for the main agents in sows with vulvar discharge (VD) and healthy (HE). Green and red lines indicate positive and negative correlation, respectively. Line thickness is proportional to the strength of the Pearson correlation.

The network analysis shows the correlations between the most influential bacterial genera in vulvar discharge and healthy sows (Fig. 8). For VD females, positive correlations were predominant between potentially pathogenic bacteria such as *Escherichia*-Shigella, *Streptococcus*, *Trueperella*, *Corynebacterium,* and *Prevotella*. For healthy sows, however, negative correlations were predominant with *Escherichia-Shigella* being an important node in these correlations, but with a significant positive correlation with *E. faecalis*.

## Discussion

Despite the great genetic advances that have made female swine hyper prolific, sow mortality has increased worldwide. Reproductive diseases are responsible for an important part of these deaths^2^, with vulvar discharge being one of the main clinical signs pointing to infectious problems in the reproductive tract of sows^1^. Few studies have evaluated the sow vaginal bacterial composition between healthy sows and sows with urogenital infection, with a limited number of animals^11,12^. To increase knowledge about the vaginal microbiota of healthy and diseased sows, and to fill the gap for this assessment in Brazilian herds, we evaluated the hypothesis that there may be some differences in the bacterial vaginal content of sows with and without purulent vulvar discharge.

The culture-dependent approach revealed a rich population of bacterial species in the vaginal canal of sows, showing that VD and HE females had a predominance of certain taxa such as *E. coli, E. faecalis*, and *Streptococcus* and *Staphylococcus* species, in agreement with previous studies of bacterial isolation from the reproductive tract of sows^4,16,17^.

We found potentially harmful bacteria in a higher frequency in VD sows. Winter et al.^18^ reproduced the vulvar discharge syndrome through uterine experimental inoculation with *S. hyicus* in swine females. This species was isolated with high frequency in our study in VD sows. *C. diphtheriae*, already described in genitourinary infection of sows and public health importance for its role in human infections^19–21^, as well as *T. pyogenes, B. pyogenes,* and *S. dysgalactiae,* also reported in reproductive disorders in cows and sows^9,22–29^, were also isolated more frequently in VD sows. But there was no statistical difference in the frequency of these agents between HE and VD sows. These agents were also associated with a high frequency in VD sows, suggesting that these associations may be important for causing disease.

However, since the culture-dependent method used does not quantify specific bacteria and may also be biased since most agents do not grow easily in culture media^30^, we performed a 16S rRNA metagenomic sequencing approach in 72 of these vaginal samples to characterize the bacterial population between VD and HE sows.

Our study showed that sows’ vaginal bacterial communities differed between herds. This can be due to several uncontrolled factors, such as nutritional, environmental, and genetic differences, hygiene level of the facilities, and microclimate. Although there are some fluctuations in relative abundance at the phylum and genus level between herds, which explains the difference in the beta-diversity index, they share most of the agents, with the phyla *Proteobacteria*, *Firmicutes,* and *Bacteroidota* and the genera *Escherichia-Shigella*, *Streptococcus*, *Enterococcus,* and *Bacteroides* were among the most relatively abundant in all herds.

Beta diversity analysis revealed a significant difference between HE and VD sows by the unweighted UniFrac PCoA analysis for two of the four herds, which indicates that the difference between bacterial communities could be in specific features^31^, such as *B. pyogenes*, *S. dysgalactiae* and *S. hyicus*, found in greater relative abundance in VD sows.

*Proteobacteria*, *Firmicutes,* and *Bacteroidetes* were the predominant phyla for both HE and VD groups. Remarkably comparable results were found by other studies in both healthy and diseased animals^7,9–12^, indicating that the vaginal microbiota of sows is dominated by these phyla. At the genus level, there was a high agreement with the culture-dependent method results, since *Escherichia, Streptococcus, Enterococcus,* and *Staphylococcus* are among the highest relative abundances. Interestingly, the phylum *Bacteroidetes* and the genus *Bacteroides* were found in high relative abundances only in the metagenomic approach, in contrast to the culture-dependent method. This may be due to the difficulty of traditional culture methods to cover fastidious bacteria, such as the obligate anaerobes *Bacteroides* members^32^. This reinforces that the metagenomic approach may be more effective in characterizing the microbiota due to its higher resolution to identify low-abundance or fastidious microbes, while culture-dependent methods can confirm the viability of the identified agents, making them complementary techniques.

The species *Acinetobacter lwoffii* was significantly more frequent in HE sows in the culture method. Luque et al.^7^ also found these genera in vaginal samples of healthy sows. As these authors point out, despite not being described as causing urogenital infection in sows, these genera have been described as carriers and spreaders of resistance genes for antimicrobials used in veterinary practice and human medicine^33,34^ and their presence requires attention.

*Bacteroides, Staphylococcus,* and *Porphyromonas*, found in higher relative abundance in VD than in HE sows, were also detected in higher relative abundance in sows with endometritis in recent studies^11,12^. Even though the sequencing did not differentiate *Staphylococcus* species, we suggest that part of this high relative abundance in VD sows may be represented by *S. hyicus*, as shown by culture and identification by MALDI-TOF MS. *S. hyicus* has been described as capable of causing uterine infection in an experimental infection. Also, *S. dysgalactiae* had a notably higher relative abundance also in VD sows. Kiefer et al.^9^ found that *S. dysgalactiae* was significantly more abundant in sows with a high score for pelvic organ prolapse, which reinforces the importance of this species.

We used the ANCOM test to perform a differential abundant analysis between specific taxa to compare VD and HE sows. Previous researchers showed that statistical methods commonly used in differential abundance analysis, such as ANOVA or Kruskal–Wallis, are not appropriate for compositional data because they inflate false discovery rates (FDR) and should be avoided^35–37^. Recent studies showed that the ANCOM test was superior in controlling FDR while retaining great statistical power^36,37^. In our study, the ANCOM test revealed that *B. pyogenes* relative abundance in VD sows is higher than in HE sows, being a marker for vulvar discharge and suggesting its importance in vulvar discharges syndromes since this agent has been identified in uterine infections in cows and sows^12,38,39^. Indeed, Zhang et al.^12^ suggest a link between the increase of some bacteria in the gut microbiota and the onset of endometritis in sows, including the *Bacteroides* genus. The pathogenesis of *B. pyogenes* infections is still unclear, especially in the swine species. But recent studies described this agent in important infections in humans, such as purulent abscesses, infected wounds, and urinary tract infections^32^. Only herd-3 showed a significant difference in the ANCOM method for the higher relative abundance of *B. pyogenes* in VD females, which could explain the difference in beta-diversity observed between the HE and VD groups for this herd. Moreover, we found that the relative abundance of *B. pyogenes* was also significantly higher in VD females for the ANCOM method when samples from all herds were analyzed together. In addtion, the graphs show that for all herds the relative abundance of *B. pyogenes* was greater in VD females. Although this difference was most notable for herd-3 and herd-4, this is an important finding across the entire dataset.

Network analysis correlations between the genera identified by metagenomic sequencing show that VD sows had more positive correlations between agents recognized as pathogenic for the reproductive tract, such as *Escherichia-Shigella* and *Trueperella,* and *Escherichia-Shigella* and *Streptococcus*^18,25–27^*.* Significantly, the association between these species also occurred in the culture-dependent method. A positive correlation between *Corynebacterium* and *Prevotella* may also be important since these agents have already been described in reproductive disorders of sows^9,20^ and women^40^. Such correlations did not occur for HE females, in which *Escherichia-Shigella* and *Enterococcus* are dominant nodes, with negative correlations, showing dominance for these agents. This is also corroborated by the MALDI-TOF MS and relative abundance graphs since *Escherichia* and *Enterococcus* present higher frequency and relative abundance in HE females in all herds. The *Enterococcus* genus also has a strong positive correlation with the *Veillonella* genus commonly found in considerable abundance in the vaginal microbiota of sows, between 2 and 6%, but without a relationship with disease^9,41^.

This study provides new evidence on the vaginal bacterial composition of sows with and without purulent vulvar discharge. Most bacteria obtained in culture were also present in the metagenomic sequencing, showing the viability of the detected agents. The vaginal microbiota of these groups shares most of the features identified by 16S rRNA sequencing, but punctual changes in the abundance of certain agents differentiated VD and HE females*,* especially *B. pyogenes*, which can be proposed as a marker for VD sows. Higher frequencies or relative abundances of specific agents in VD sows, and the association between them, also require attention. Evidence suggests that dysbiosis in the vaginal canal of female swine that presents purulent vulvar discharge occurs by the association and increase of some potentially pathogenic bacteria such as *B. pyogenes, S. dysgalactiae, S. hyicus, C. diphtheriae,* and *T. pyogenes.*

## Methods

### Animals and sample collection

Three hundred ten vaginal samples were collected from commercial crossbred sows housed in four commercial farms in different Brazilian states: São Paulo, Minas Gerais, Paraná, and Mato Grosso. Two hundred seventy samples were collected from sows presenting purulent vulvar discharge (VD) and 40 were collected from healthy sows (HE).

Animals were inspected for purulent vulvar discharge with the use of a sterile and disposable vaginal speculum during pregnancy or in the lactation period, at least three days after the farrowing process, combined with the observation of the presence of purulent material on the floor and in the bars below the female. The introduction of the speculum was always performed by two veterinarians, one dealing with the vulva and exposing the vaginal canal and the other introducing the speculum to avoid external contamination Samples were only collected if purulent secretion was present in the deep region of the vaginal canal, after the passage of the speculum, which also excludes the possibility that this purulent material originates from vulvar lesions or vaginitis. For the collection, two sterile swabs (one for culture and one for metagenomic approaches) were collected, placed in transport media, and immediately refrigerated until arrival at the laboratory for further processing. Secretions considered to be normal-appearing and lochia were not collected. Only secretions very suggestive of infectious processes, intensely purulent and with lumps were collected and were considered in further analyses. Sows that had just urinated were not collected. If antimicrobials were used in any of the animals in the previous 30 days before collection, these females were not included in further analysis. Healthy females were collected if they did not present vulvar discharge, hyperthermia, low body condition score, had been medicated, or had any other disease condition.

### Ethics approval

This study was evaluated and approved by the Animal Use Ethics Committee (CEUA) of the Faculty of Veterinary Medicine and Animal Science of the University of São Paulo, Brazil (CEUA Process Number 1875170317). This Committee follow all international guidelines and regulations to evaluate studies and experiments on live vertebrates. The authors declare that the methods are in accordance with ARRIVE guidelines (https://arriveguidelines.org).

### Bacterial culture

Swabs were plated on MacConkey agar, Chromagar Orientation®, and Columbia agar with 5% sheep blood (Difco-BBL, Detroit, MI /USA), and the plates were incubated at 37ºC for 24 to 48 h under aerobic conditions. For anaerobic growth, swabs were seeded on *Brucella* agar with 5% sheep blood supplemented with hemin and menadione (Difco-BBL, Detroit, MI, USA) and incubated at 37ºC for 24 to 48 h. After growth, the isolated bacterial colonies were transferred to 3.0 ml of BHI broth (Brain Heart Infusion – Difco), and from this culture, aliquots were separated for storage at -80º C and for protein extraction for identification by Matrix Associated Laser Desorption Ionization Time-of-Flight mass spectrometry (MALDI-TOF MS).

### Identification of bacterial strains by MALDI-TOF MS

For bacterial identification by MALDI-TOF MS, the colonies were subjected to ribosomal protein extraction using the protocol described by Hijazin et al.^42^. The Microflex™ mass spectrophotometer (Bruker Daltonik) from the São Paulo State Environmental Company (CETESB) was used. To capture the protein spectra, 1.0 µl of protein suspension was transferred to a 96-well stainless-steel plate and, after drying at room temperature, 1.0 µl of the polymer matrix (α-cyano-4-hydroxycinnamic acid) was added. Each strain was distributed in three wells and for each plate, two readings were performed with the FlexControl™ software (Bruker Daltonik) using the MTB_autoX method. Finally, the BioTyper™ 3.0 software (Bruker Daltonik) compared the captured spectra for each strain with the manufacturer's library for bacterial identification. By comparing the presence/absence of specific peaks, a log (score) value was obtained. The criteria for interpreting the standards used in this study were those of the manufacturer Bruker Daltonik: scores ≥ 2.0 were accepted for species attribution, and scores ≥ 1.7 and < 2.0 were used only for genus identification.

### DNA extraction and 16S rRNA gene sequencing

For the metagenomic approach, 72 swabs (40 VD and 32 HE) had their bacterial DNA extracted using the DNeasy® Blood & Tissue Kit (QIAGEN), following the manufacturer's recommendations. DNA concentration and integrity were verified in the NanoDrop® 2000 equipment and by agarose gel electrophoresis before being sent to the sequencing facility. Briefly, 16S rRNA amplicons were produced using specific primers for the 16S-V4 region (515F-806R) along with barcodes. All PCR reactions were conducted with Phusion® High-Fidelity PCR Master Mix (New England Biolabs). The mixed PCR products were purified with Qiagen Gel Extraction Kit (Qiagen, Germany) and the libraries generated with NEBNext® UltraTM DNA Library Prep Kit for Illumina and quantified via Qubit and qPCR. Amplicons were sequenced on the Illumina MiSeq platform to generate 250 bp paired-end reads.

### Bioinformatics analysis

Downstream analyses were performed using the QIIME 2 platform^43^. Paired-end reads were assigned to samples based on their unique barcodes and truncated by cutting off the barcode and primer sequences with the cutadapt tool^44^ and the union of reads was performed with the Vsearch plugin^45^. A quality filter was applied with the quality-filter plugin by the q-score-joined method^46^ and a denoising step was performed using the deblur plugin with the denoise-16S method^47^. Sequences with ≥ 97% or more similarity were assigned to the same OTU (Operating Taxonomic Unit) with the Vsearch plugin^45^ and were taxonomically classified using a pre-trained classifier trained on the SILVA database^48,49^.

### Statistical analyses

Fisher's exact test was used to compare the frequencies of the main agents isolated in the culture method and identified by MALDI-TOF MS, between herds and between HE and VD sows, under the significance level of 5%. For statistical analysis of alpha diversity comparing the herds and groups of healthy females and those with purulent vulvar discharge, the Kruskal–Wallis test was performed, with a significance level of 5%. For the comparison of beta diversity, the permutational multivariate analysis of variance (PERMANOVA) was performed with 999 permutations. A significance level of 5% for false discovery rate adjusted *p*-value (*q*-value) was considered. Differential abundance analysis was performed using the ANCOM^37^ test on the QIIME 2 platform^43^. Figures and graphics were performed with the qiime2R package^50^ and ggplot2 package^51^ in the R-Studio software^52^.

For network analysis, the absolute abundance data were submitted to non-paranormal transformation according to Liu et al.^53^ to build a correlation matrix based on Pearson correlation. Plots were generated using the qgraph package^54^.

## Supplementary Information

Supplementary Information 1.

Supplementary Information 2.

Supplementary Information 3.

## Data Availability

The targeted metagenomic dataset was deposited in the NCBI’s Sequence Read Archive (SRA) database under the accession code PRJNA773131.
