# Supplementary material for: Vaginal microbiota signatures in healthy and purulent vulvar discharge sows
Source: Sci Rep. 2022 Jun 1;12:9106. doi: 10.1038/s41598-022-13090-8 (PMC9160009; doi:10.1038/s41598-022-13090-8)
Supplement: Supplementary file 1 — Supplementary Information 1. [file 41598_2022_13090_MOESM1_ESM.docx]

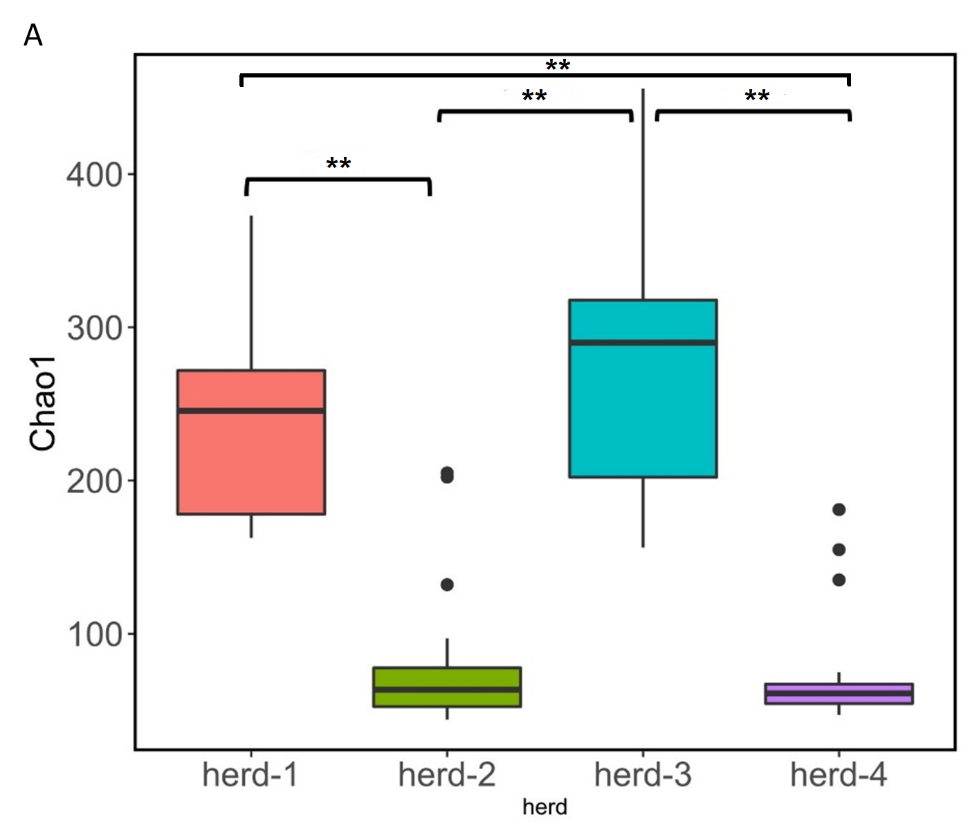

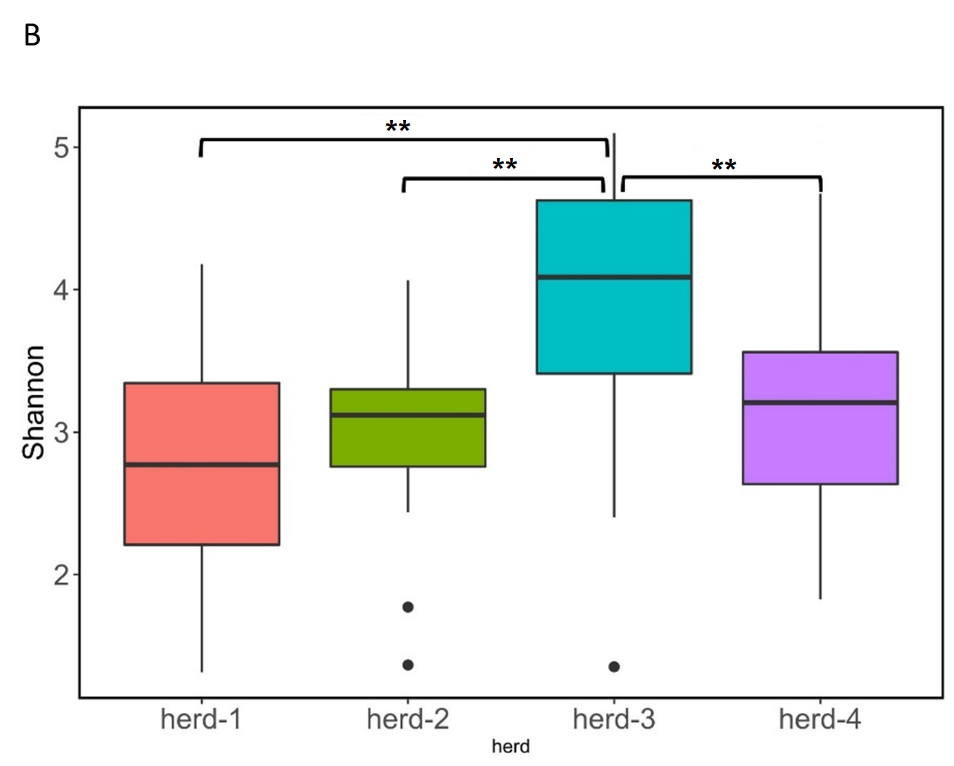


**Figure S1:** Alpha diversity box-plots for (A) Chao1 and (B) Shannon indices for the four herds from sows where sows were sampled. ** Indicates significant differences between herds (Kruskal-Wallis test, *p* < 0.01).


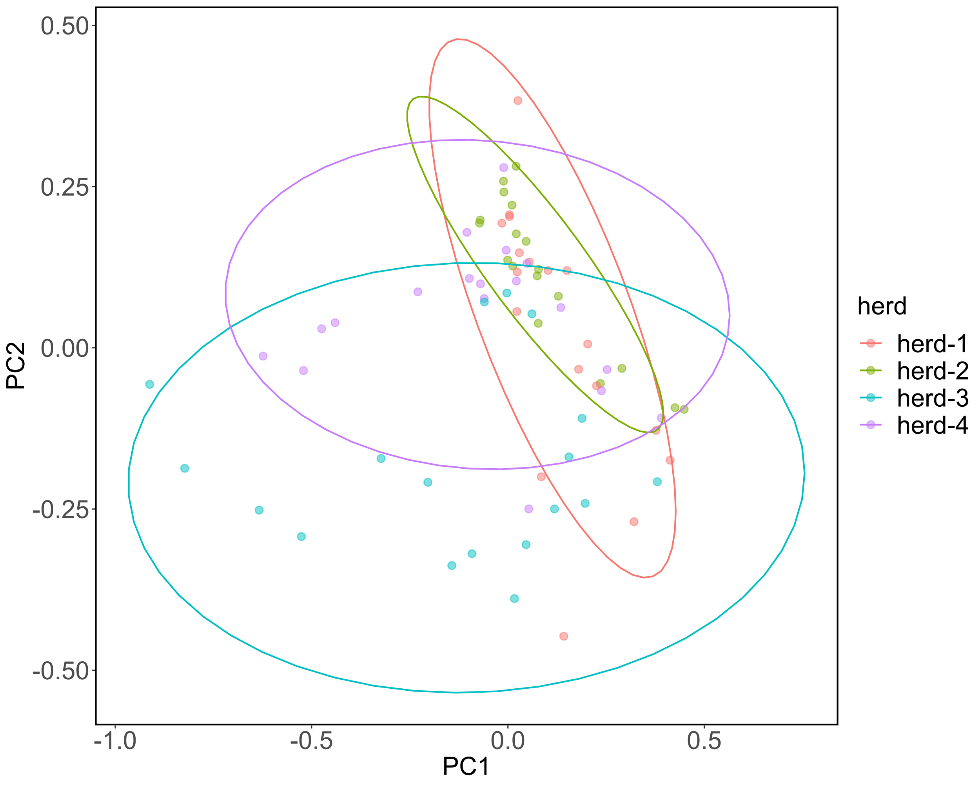


**Figure S2**: Weighted UniFrac Principal Coordinates Analysis (PCoA) between herds. Herd-1 and herd-2 were not different from each other, but differences were observed between herd-1 vs. herd-3; herd-1 vs. herd-4; herd-2 vs. herd-3; herd-2 vs. herd-4 and herd-3 vs. herd-4 (PERMANOVA*, p* < 0.05*; q* < 0.05)

**Table S1:** Fisher’s Exact Test *p*-value between herds for the main agents and between VD vs. HE sows for MALDI-TOF identification data.

| **Agent** | **Fisher’s Exact Test *p-value* between herds** | **Fisher’s Exact Test *p-value* between VD and HE sows** |
| --- | --- | --- |
| *Streptococcus suis* | 0.76 | 0.05 |
| *Staphylococcus hyicus* | 0.27 | 0.09 |
| *Streptococcus dysgalactiae* | 0.81 | 0.09 |
| *Escherichia coli* | 0.30 | 0.06 |
| *Enterococcus faecalis* | 0.42 | 0.02 |
| *Rothia nasimurium* | 0.45 | 0.18 |
| *Streptococcus hyovaginalis* | 0.09 | 0.04 |
| *Acinetobacter lwolffii* | - | 0.04 |
